# Chronic lymphatic leukaemia in a worker with a long-term occupational exposure to petroleum product mixtures: a case report

**DOI:** 10.2478/aiht-2026-77-4094

**Published:** 2026-03-30

**Authors:** Diana Bućan, Ivan Bućan

**Affiliations:** Occupational Health Practice, Split, Croatia; University of Split, School of Medicine, Split, Croatia

**Keywords:** benzene, haemoblastosis, myelogram, occupational disease, petrol station worker, benzen, hemoblastoza, mijelogram, profesionalna bolest, radnik na benzinskoj postaji

## Abstract

Following a regular occupational health checkup, a 63-year-old worker employed at petrol station for thirty years was diagnosed with chronic lymphocytic leukaemia (CLL). The patient’s occupational history revealed long-term daily exposure to petroleum-derived vapours. As part of a routine occupational health assessment, he underwent comprehensive laboratory testing, followed by further evaluation and treatment by a haematologist, including a myelogram, immunophenotyping of haemoblastosis, and bone marrow histopathology. Based on the completed diagnostic workup, the patient was diagnosed CLL with B-cell predominance. Due to the nature of the disease and job demands, the worker was declared permanently unfit for work at his current workplace. The diagnosis was recognised as an occupational disease by the Croatian Health Insurance Fund thanks to the expert opinion, issued by the occupational health specialist, who, after an extensive toxicological assessment and the exclusion of other possible causes, linked 30 years of occupational inhalation exposure to petroleum products containing benzene with the onset of this malignant disease. This case highlights systemic shortcomings in the assessment of occupational exposure to mixtures of petroleum products, inhalation of vapours, and potential dermal contact among petrol station workers, deficiencies in occupational safety measures, and the crucial role of occupational medicine specialists in the prevention, early detection, and risk management before disease progression, as well as in supporting the legal and occupational rights of affected workers.

## Abstract (hr)

Prikazan je slučaj 63-godišnjeg radnika koji je trideset godina neprekidno radio kao prodavač na benzinskoj postaji, a kojem je nakon preventivnog liječničkog pregleda dijagnosticirana kronična limfocitna leukemija. Pacijent je imao radnu anamnezu dugotrajne svakodnevne izloženosti udisanju para naftnih derivata. U sklopu rutinskog pregleda medicine rada podvrgnut je kompletnim laboratorijskim pretragama, a zatim pregledu i liječenju kod hematologa s mijelogramom, imunofenotipizacijom hemoblastoze i punkcijom koštane srži s histopatološkim nalazom. Na temelju urađene dijagnostike postavljena je dijagnoza kronične limfocitne leukemije s predominacijom B limfocita. Zbog prirode bolesti i zahtjeva radnog mjesta, radnik je ocijenjen trajno nesposobnim za rad na ugovornom radnom mjestu. Zahvaljujući ekspertnom mišljenju specijalista medicine rada i sporta Instituta za medicinska istraživanja i medicinu rada (Zagreb), dobivenom na osnovi detaljne kliničko-toksikološke analize, koje je upozorilo na profesionalnu etiologiju zloćudne bolesti prouzročene 30-godišnjim radom u inhalacijskoj izloženosti naftnim derivatima sa sadržajem benzena, Hrvatski zavod za zdravstveno osiguranje priznao je dijagnozu kao profesionalnu bolest. Ovaj slučaj ističe sustavne propuste u procjeni profesionalne izloženosti radnika na benzinskoj postaji (smjese naftnih derivata; udisanje para i mogući dermalni kontakt), nedostatke zaštite na radu te ključnu ulogu specijalista medicine rada u prevenciji, ranom otkrivanju, smanjenju rizika od progresije bolesti u uznapredovali stadij i ostvarivanju prava oboljelog radnika.

Workers at petrol stations face many health risks due to occupational exposure to petroleum products derived from crude oil and natural gas. These include volatile organic compounds (VOCs) like benzene, toluene, xylene, and *n*-hexane, generally recognised as hazardous (1,2,3).

Several recent international studies (4,5,6) report health issues among petrol station workers and agree that an important practice for efficient risk management would be to lower exposure to the above noxae. Available engineering measures would include ventilation systems at petrol stations, petrol vapour recovery systems, leak detection, and gas monitoring for benzene, H_2_S, and VOCs. Exposure can further be reduced with the appropriate personal protective equipment (PPE), including chemical-resistant gloves (nitrile, butyl), flame-resistant clothing, eye protection, and respirators when indicated (7,8,9).

Chronic exposure to high levels of VOCs is associated with narcosis, arrhythmia, and chemical pneumonitis (4, 6, 10), chronic skin contact with oils and fuels with dermatitis and eczema (4, 11), chronic exposure to *n*-hexane with peripheral neuropathy (1, 2, 4), chronic exposure to solvents with memory problems, mood changes, and impaired cognitive function (4, 12), and finally, chronic exposure to benzene with haematotoxicity and the onset of aplastic anaemia, leukopenia, and increased risk of leukaemia (2, 4, 6, 13). In fact, the International Agency for Research on Cancer (IARC) classifies not only benzene but also diesel engine exhaust as carcinogenic to humans (IARC Group 1), while gasoline exhaust is classified as possibly carcinogenic to humans (Group 2B) (14, 15).

Here we report a case of a worker who developed chronic lymphocytic leukaemia (CLL) over thirty years of work at a petrol station. Our aim is to raise the issue of proving that such diagnosis is owed to continuous, long-term exposure to mixtures of petroleum-derived substances despite the lack of exposure data, which is the consequence of deficient occupational safety measures or failure to conduct environmental monitoring at high-risk workplaces. In view of these dilemmas, we recommend a set of actions for an occupational health specialist to take in such cases: from suspicion of the disease, specialist referral, and early diagnosis to establishing an occupational disease and supporting the worker’s claim to legal remedy.

## CASE PRESENTATION

The patient is a 63-year-old man, a heavy motor vehicle driver by profession, with completed secondary vocational education. His total work experience amounts to approximately 36 years, of which he spent the first five working as a driver and the rest (since 1989) as a petrol station attendant. He had worked rotating shifts in open and enclosed environments involving exposure to petroleum vapours, derivatives, and exhaust gases. His main tasks were filling vehicles with fuel, vehicle washing service, washing windows, checking tyre pressure, refilling water, antifreeze and oil in vehicles, arranging the sales area, keeping sales and stock records, and monitoring/controlling the operation of installations, equipment, and devices. While working at gas stations in Split-Dalmatia County, he changed three locations, each involving workload spikes during the tourist season. The patient also reported sampling fuel from tank trucks and emphasised that vapour recovery valves were not installed until 2012. A review of the available documentation showed that no measurements of harmful substances were carried out at his workplace nor was fuel vapour exposure risk assessed. Also, there are no records indicating consistent use of personal protective equipment or evidence of worker training regarding occupational risks.

As part of regular check-ups, the worker was referred by the employer to occupational medicine specialist. During the examination, the patient reported constant fatigue and night sweats over the preceding months. He was undergoing treatment for degenerative changes of the lumbosacral spine, with radiologically confirmed changes at the L4/L5 segment. He had a history of chronic gastritis and post-traumatic stress disorder (owed to combat service in the Croatian Homeland War in the 1990s). Medical records showed no treatment for severe acute or chronic disorders. He had not smoked since 1982 and denied any allergies. He also denied any family history of malignancies.

## INVESTIGATIONS

Following a detailed medical history and physical examination, the patient underwent a comprehensive laboratory evaluation. Initial biochemical tests (total proteins, albumin, globulins, total, direct and indirect bilirubin, transaminases, total cholesterol, triglycerides, high- and low-density lipoproteins) and urinalysis were within reference ranges. The complete blood count revealed leukocytosis (27.2×10^9^/L; reference range: 3.4–9.7×10^9^/L) and lymphocytosis (22.71×10^9^/L or 83.5 %; reference range: 1.0–4.8×10^9^/L or 20–46 %), which raised suspicion of an underlying haematological disorder.

Given the occupational risks, these findings warranted further haematological evaluation, including a myelogram, immunophenotyping of haemoblastosis, and histopathological analysis of a bone marrow sample. The myelogram revealed high levels of lymphoid cells, predominantly small lymphocytes (48 % of total cells) with occasional lymphoplasmacytoid and atypical forms, while immunophenotyping revealed that the lymphocyte population was dominated by B cells with the following phenotypes: CD45^+^, CD19^+^, CD20^+^, CD22^+^, CD79b^−^, CD5^+^, CD23^+^, CD11c^+^, CD38^−^, CD10^−^, and CD138^−^ with no demonstrable surface or cytoplasmic immunoglobulin light chain expression, which is consistent with B-cell chronic lymphocytic leukaemia (B-CLL). Biopsied bone marrow revealed an interstitial pattern and small aggregates of up to 30 % of CD20^+^, CD5^+^, and ZAP-70-negative small lymphoid cells. Approximately 10 % of the cells were CD3-positive small lymphoid cells, which also pointed to CLL.

After the diagnosis, the haematologist took the “watch-and-wait” approach (23, 24) with active surveillance consisting of quarterly consultations and laboratory monitoring (complete and differential blood count, biochemistry, urinalysis). Over the twelve-year of follow-up, no changes were observed that would require medical treatment. Leukocyte counts ranged from 19.6×10^9^/L to 76.5×10^9^/L, while lymphocyte counts ranged from 17.35×10^9^/L to 51.61×10^9^/L (82.7–92.7 %). The patient had mild B-CLL symptoms (fatigue and occasional night sweats), while haematological findings showed no disease progression with symptoms of anaemia, thrombocytopenia, lymphadenopathy, or rapid lymphocyte doubling time.

## OCCUPATIONAL DISEASE AND DISABILITY STATUS

Based on the patient’s occupational and medical history, submitted medical documentation, and the opinion of occupational health specialist, the Croatian Health Insurance Fund recognised the diagnosed CLL as occupational disease. The key supporting documentation was the expert opinion issued by the Institute for Medical Research and Occupational Health, Zagreb, Croatia in 2020, which associated the onset of the CLL with 30 years of occupational exposure to petroleum products containing benzene.

This expert clinical-toxicological opinion was based on the following facts: the patient was exposed to daily inhalation of petroleum product vapours for 30 years, especially during filling and sampling fuel. Petroleum products handled by the patient contained benzene, which is classified as a Group 1A carcinogen by the European Chemicals Agency (ECHA), and as a Group 1B carcinogen by the EU Regulation on Classification, Labelling and Packaging of hazardous chemicals (hazard symbol H350 - may cause cancer; H340 - may cause genetic damage) (16). In addition, the International Agency for Research on Cancer (IARC) classifies benzene as a Group 1 carcinogen (“human carcinogen”). According to earlier reports (17), its concentration range in petroleum products is 1–5 %.

Exposure to benzene leads to malignant proliferation of the bone marrow and the development of leukaemia and other related diseases, including chronic lymphocytic leukaemia (18, 19). The symptoms of these malignant disorders usually occur after a longer period of latency.

Considering that benzene is a genotoxic carcinogen for which a definitive limit value without carcinogenic effects cannot be proven and considering the fact that the permissible limit values for occupational exposure to benzene have been significantly lowered since the 1990s thanks to accumulated knowledge about its toxicity, the possibility of a causal relationship between occupational exposure and the occurrence of CLL in this specific case cannot be ruled out. The expert opinion was also supported by many studies that associated exposure to benzene in workers working at petrol stations with the occurrence of haematological malignant diseases (19,20,21,22,23,24).

In the process of establishing entitlement to disability pension benefits, and upon the recommendation of the patient’s family physician, the patient was assessed as having completely lost work ability and was granted the right to a disability pension.

## DISCUSSION AND CONCLUSION

This case highlights the issue of missing records of occupational safety measures at the workplace (petrol station), absence of risk assessments specific to fuel vapour exposure, and undocumented implementation of personal protective equipment, which complicates establishing occupational aetiology of CLL and patient’s long-term occupational exposure to harmful compounds (e.g. benzene). Thanks to the joint approach to the case by an occupational medicine specialist, a haematologist, a forensic occupational medicine expert and a toxicologist, however, the described case was successfully resolved in favour of the patient.

In cases of long-term occupational exposure to carcinogens such as benzene, changes in the legislative framework that have a strong impact on proving cause-and-effect relationships should also be considered.

Over the working years of the patient presented here, the permissible exposure limit values for benzene have changed significantly, from 15 ppm in older ordinances, issued before 1990, to 5 ppm in 1993, and finally to the current 1 ppm, or 3.25 mg/m^3^ (25).

Since benzene is a genotoxic carcinogen for which a definitive limit value without carcinogenic effects cannot be proven, in its 2018 report of the Committee for Risk Assessment the European Chemicals Agency (ECHA) proposed to reduce the exposure limit of benzene to only 0.05 ppm, based on genotoxic effects determined in workers exposed to concentrations of 1 ppm (26). Nowadays, world policies towards the limit value of benzene are constantly being adjusted, with the USA and some European countries such as Germany advocating even further lowering of the occupational exposure limit values (OELs).

Although exposure to benzene and other volatile organic compounds in the European Union and Croatia is subject to OELs, the available documentation of the worker whose case is presented here contains no data on the measurements of benzene or other volatile compound air concentrations at the workplace nor on biological monitoring of specific exposure biomarkers. Such deficiencies may have resulted in increased cumulative exposure and contributed to the risk of developing the patient’s malignant disease.

The course of CLL is typically indolent but characterised by continuous and progressive accumulation of malignant lymphocytes in the peripheral blood and bone marrow. The consequences of this condition include immunosuppression, bone marrow dysfunction, and infiltration of organs by malignant cells. Clinical symptoms, such as fatigue, night sweats, loss of appetite and body weight, infections, lymphadenopathy, and lower back pain, develop gradually (27). Even though the exact aetiology of the patient’s CLL remains unknown, risk factors such as advanced age, male sex, years of work at the petrol station, and likely but unconfirmed exposure to petroleum product mixtures containing benzene and heavy solvents in our patient point to occupational disease.

This case report highlights the important role of the occupational medicine specialist in the systematic monitoring of workers’ health from the moment occupational hazards are identified, referring the patient to a haematologist, and the implementation of all necessary procedures to ensure documented recognition of workers’ rights.

The outlined sequence of procedures illustrates good practice for future similar cases. The occupational medicine specialist played a pivotal role in: 1) identifying suspicious findings during periodic medical examinations; 2) referring the worker for further haematological evaluation; 3) documenting occupational exposure, including consultation with toxicology experts or occupational medicine forensic experts; 4) initiating the process of recognition of an occupational disease by the Croatian Health Insurance Fund; and 5) providing guidance regarding permanent unfitness for work involving exposure to fuel vapours and the realisation of rights under disability insurance.

More importantly this case points to serious deficiencies in the occupational safety system within specific work environments, where exposure to carcinogens is possible, like in our case, the unavailability of exposure monitoring (air measurements), inconsistent use of personal protective equipment, and the absence of regular periodic risk assessments. For workplaces involving exposure to mixtures of petroleum products, the following measures are essential: 1) regular measurement of hazardous substance concentrations and biological monitoring when indicated; 2) up-to-date risk assessments and technical source-control measures; 3) consistent worker training and use of personal protective equipment; and 4) systematic preventive health surveillance with clearly defined referral protocols.

## References

[1] Chaiklieng S, Suggaravetsiri P, Autrup H (2019). Risk assessment on benzene exposure among gasoline station workers. Int J Environ Res Public Health.

[2] Tunsaringkarn T, Siriwong W, Rungsiyothin A, Nopparatbundit S (2012). Occupational exposure of gasoline station workers to BTEX compounds in Bangkok, Thailand. Int J Occup Environ Med.

[3] IARC (2012). Monographs on the Evaluation of Carcinogenic Risks to Humans.

[4] Ekpenyong CE, Asuquo EA (2017). Recent advances in occupational and environmental health hazards of workers exposed to gasoline compounds. Int J Occup Med Environ Health.

[5] Cezar-Vaz MR, Rocha LP, Bonow CA, Santos da Silva MR, Vaz JC, Cardoso LS (2012). Risk perception and occupational accident: A study of gas station workers in Southern Brazil. Int J Environ Res Public Health.

[6] Giardini I, da Poça KS, da Silva PVB, Andrade Silva VJC, Cintra DS, Friedrich K, Geraldino BR, Otero UB, Sarpa M (2023). Hematological changes in gas station workers. Int J Environ Res Public Health.

[7] Tongsantia U, Chaiklieng S, Suggaravetsiri P, Andajani S, Autrup H (2021). Factors affecting adverse health effects of gasoline station workers. Int J Environ Res Public Health.

[8] Adekunle ED, Alex OT, Adedayo OJ (2023). Knowledge of occupational hazards and safety practices among petrol station workers in Ibadan Metropolis, Oyo State, Nigeria. J Mater Sci Res Rev.

[9] Adekunle ED, Alex OT, Adedayo OJ (2023). Occupational health hazards among petrol station workers in Ibadan, Oyo State, Nigeria. Asian J Chem Sci.

[10] Adeniyi BO (2014). Pulmonary function and symptoms among petrol pump attendants in Nigeria. Int J Biol Med Res.

[11] Khoshakhlagh AH, Mohammadzadeh M, Gruszecka-Kosowska A (2024). Dermatitis, a nightmare for those exposed to environmental pollutants. J Hazard Mater Adv.

[12] Letellier N, Choron G, Artaud F, Descatha A, Goldberg M, Zins M, Elbaz A, Berr C (2020). Association between occupational solvent exposure and cognitive performance in the French CONSTANCES study. Occup Environ Med.

[13] Frolayne M, Wallace C, Zhang L, Martyn TS, Rader G, Steinmaus C (2015). In utero, and early-life exposure to benzene and the risk of childhood leukemia: A meta-analysis. Am J Epidemiol.

[14] IARC Monographs on the Evaluation of Carcinogenic Hazardss to Humans.

[15] IARC Monographs on the Identification of Carcinogenic Hazards to Humans.

[16] EUR-Lex Regulation (EC) No 1272/2008 of the European Parliament and of the Council of 16 December 2008 on classification, labelling and packaging of substances and mixtures, amending and repealing Directives 67/548/EEC and 1999/45/EC, and amending Regulation (EC) No 1907/2006 [displayed 13 March 2026].

[17] Tuomi T, Veijalainen H, Santonen T (2018). Managing exposure to benzene and total petroleum hydrocarbons at two oil refineries 1977–2014. Int J Environ Res Public Health.

[18] Watson HG, Culligan DJ, Manson LM, Ralston SH, Penman ID, Strachan MWJ, Hobson RP (2022). Hematologija i transfuzijska medicina [Haematology and transfusion medicine, in Croatian]. Davidsonove osnove interne medicine [Davidson’s Principles and Practice of Medicine, in Croatian].

[19] Khalade A, Jaakkola MS, Pukkala E, Jaakkola JJK (2010). Exposure to benzene at work and the risk of leukemia: a systematic review and meta-analysis. Environ Health.

[20] Stenehjem JS, Kjærheim K, Bråtveit M, Samuelsen SO, Barone-Adesi F, Rothman N, Lan Q, Grimsrud TK (2015). Benzene exposure and risk of lymphohaematopoietic cancers in 25 000 offshore oil industry workers. Br J Cancer.

[21] Vlaanderen J, Lan Q, Kromhout H, Rothman N, Vermeulen R (2011). Occupational benzene exposure and the risk of lymphoma subtypes: a meta-analysis of cohort studies incorporating three study quality dimensions. Environ Health Perspect.

[22] Smith MT, Zhang L, McHale CM, Skibola CF, Rappaport SM (2011). Benzene, the exposome and future investigations of leukemia etiology. Chem Biol Interact.

[23] Chaiklieng S, Tongsantia U, Suggaravetsiri P, Autrup H (2024). Altered haematological parameters in gasoline station workers due to benzene exposure. Safety.

[24] Binsaleh NK, Eltayeb R, Bashir EM, Idris HME, Althobiti MM, Ahmed HG, Khan MWA, Qanash H (2024). Insight into hematological parameters of petrol station workers. Eur Rev Med Pharmacol Sci.

[25] Pravilnik o zaštiti radnika od izloženosti opasnim kemikalijama na radu, graničnim vrijednostima izloženosti i biološkim graničnim vrijednostima [Ordinance on the protection of workers from exposure to hazardous chemicals at work, exposure limit values, and biological limit values, in Croatian].

[26] ECHA RAC recommends an occupational exposure limit for benzene [displayed 13 March 2026].

[27] Leukaemia Foundation Chronic lymphocytic leukaemia (CLL) [displayed 13 March 2026].

